# The Gustave Roussy Immune score (GRIm score) as a novel prognostic score for early breast cancer patients: A real-world retrospective study

**DOI:** 10.7150/ijms.99724

**Published:** 2024-10-14

**Authors:** Chunlei Tan, Jinling Xu, Xiaotian Yang, Danping Wu, Shiyuan Zhang, Shuqiang Liu, Boqian Yu, Yuanxi Huang

**Affiliations:** 1 Department of Breast Surgery, Harbin Medical University Cancer Hospital, Harbin, Heilongjiang 150081, P.R. China.; 2 Endoscope Department, Harbin Medical University Cancer Hospital, Harbin, Heilongjiang 150081, P.R. China.

**Keywords:** breast cancer, Gustave Roussy immune score, NLR, lactate dehydrogenase, prognosis

## Abstract

**Background and objective:** The aim of this research is to investigate whether the GRIm score serves as a novel prognostic tool for predicting the survival rates among early breast cancer patients undergoing surgical treatment.

**Methods:** This retrospective study included 313 cases of breast cancer patients hospitalized in our hospital from January 2015 to November 2015. All enrolled patients received surgery and had no metastasis. The GRIm score was based on five objective markers: (1) albumin level (<3.5 g/L = 1 point), (2) LDH level (≥245 U/L = 1 point); (3) AST-to-ALT ratio (≥1.44 = 1 point); (4) total bilirubin level (≥21 μmol/ml = 1 point); (5) NLR (≥1.51 = 1 point). The best critical value was 1.51 for NLR by ROC. Patients were categorized into two groups based on GRIm scores: low-score group (0 point) and high-score group (1 to 5 points). Kaplan-Meier method and log rank test were utilized to estimate disease free survival (DFS) and overall survival (OS). Both univariate analysis and multivariate Cox analysis were used to analyze the relationship among the enrolled parameters. Nomograms were formulated reliant on the outcomes of multivariate Cox analysis.

**Results:** Based on the GRIm score, the cohort was divided into two groups: a low-score group with 81 cases and a high-score group with 232 cases. The mean DFS and OS were significantly prolonged in low-score group compared to high-score group (DFS: 74.39 vs. 66.20 months, χ^2^=8.729, P=0.0031; OS: 83.71 vs. 76.40 months, χ^2^=8.729, P=0.0031). According to multivariable analysis, GRIm score was notably correlated with DFS (HR: 2.789, 95% CI: 1.304-5.965, P= 0.004) and OS (HR: 3.015, 95% CI: 1.409-10.087, P=0.004). Nomograms exhibited excellent predictive performance for DFS (C-index: 0.823) and OS (C-index: 0.807).

**Conclusions:** GRIm score serves as a predictive tool for assessing the prognosis of early breast cancer patients. Nomograms based on GRIm score show good prediction ability.

## Introduction

Traditionally, the main treatments for breast cancer patients include surgery and chemotherapy based on anthracyclines and taxanes, which results in the recovery rates varying by clinical stage and subtype 1. Although the prognosis of breast cancer patients has been gradually improved by adding endocrine therapy or radiotherapy on the basis of cytotoxic chemotherapy, breast cancer remains the leading cause of cancer-related deaths among women globally 2. In the past 10 years, the new combination treatments for breast cancer, such as the combination of cyclin-dependent kinase (CDK) 4/6 inhibition and endocrine therapy 3, standard chemotherapy in conjunction with immunotherapy 4, have significantly improved the prognosis of breast cancer patients.

Recently, many studies have reported the relationship between nutrition, inflammation, immunity, and tumor development 5-8. At present, the hematological examinations commonly used in clinic include blood routine, biochemical tests, coagulation function tests, and tumor marker tests. A variety of scoring systems used to guide the prognosis of clinical trials and patient selection divide patients into different prognostic risk groups by using the combination of clinical and laboratory parameters based on blood routine or/and biochemical tests 7,9-11. In recent years, with the increasing application of immunotherapy in the field of oncology, new scoring systems (such as MD Anderson immune checkpoint inhibitor score and Gustave Roussy immune score) have been developed and constructed to evaluate patient selection in clinical trials of immune checkpoint inhibitors 12,13. Gustave Roussy Immune score (GRIm score) was firstly reported by Bigot *et al.*, based on lactate dehydrogenase (LDH) level, albumin level, total bilirubin level, AST-to-ALT ratio, and neutrophil to lymphocyte ratio (NLR). This scoring system was developed in some malignant tumors, such as advanced non-small cell lung cancer (aNSCLC), metastatic colorectal cancer (mCRC), advanced pancreatic adenocarcinoma (PDAC), undergoing immune checkpoint inhibitors to ascertain patients who may respond favorably to the current treatment 14. These findings indicate that the GRIm score may be a valuable prognostic marker for cancer patients, which clinical doctors can use to stratify patients and develop personalized treatment plans. However, the representativeness of breast cancer patients is insufficient in the discovery and validation cohorts by this scoring system. The role of this prognostic score to forecast the survival outcome among breast cancer patients is still uncertain. Hence, the aim of our study was to investigate whether the GRIm score determines the prognosis, and to provide practical guidance for breast cancer patients who have undergone surgery.

## Methods

### Patients

The 313 cases of breast cancer patients who received operations in our Hospital from January 2015 to November 2015 were enrolled in this retrospective cohort study. The demographic, clinical, and pathological data were gathered retrospectively from the electronic medical records of each enrolled patient. This research received approval from the Ethics Committee of Harbin Medical University Cancer Hospital (Grant Number: KY2023-38). In this study, all participants were thoroughly informed about the objectives, procedures, potential risks, and their rights related to the research. Specifically, written informed consent was obtained from each participant, documenting their agreement to participate in the research and authorizing the use of their data for scientific analysis.

Based on the histological examination, a histological diagnosis of breast cancer was confirmed for all enrolled patients. The inclusion criteria were: 1) Blood routine, biochemical examination, coagulation function, and tumor marker examination one week before surgery; 2) Complete medical records, and follow-up information; 3) Without distant organ metastasis. Exclusion criteria were: 1) Suffering from metastatic tumor or other malignant tumors; 2) Preoperative chemotherapy or radiotherapy; 3) Accompanied by underlying diseases that were difficult to control, and could not be treated surgically.

### Definition of GRIm score

For the first time, the GRIm score was calculated as outlined in Bigot's study 14. In the current study, the GRIm score was based on five objective markers:(1) albumin level (<3.5 g/L = 1 point, ≥3.5 g/L = 0 point); (2) LDH level (<245 U/L = 0 point, ≥245 U/L = 1 point); (3) AST-to-ALT ratio (<1.44 = 0 point, ≥1.44 = 1 point); (4) total bilirubin level (≥21μmol/ml = 1 point, <21μmol/ml = 0 point); (5) NLR (≥1.51 = 1 point, <1.51 = 0 point). The calculation of the AST-to-ALT ratio involved dividing the serum level of Aspartate aminotransferase (AST) by the serum level of Alanine aminotransferase (ALT). The baseline peripheral neutrophil count was divided by the lymphocyte count prior to surgery to calculate the NLR. In our cohort, the best critical value for NLR by ROC with the highest sensitivity and specificity to predict OS. And the best critical value for NLR was 1.51 in this study. Patients were categorized into two groups based on their GRIm scores: the low-score group (0 point) and the high-score group (ranging from 1 to 5 points).

### Followed-up and statistical methods

The disease-free survival (DFS) referred to the duration between the date following curative resection and the occurrence of either local or distant metastasis. The overall survival (OS) was defined as the duration that begins on the date following curative resection and ends either with the death of the patient for any reason or at the date of the last follow-up, depending on the context. All statistical analyses were conducted using the SPSS Statistics software version 22.0, provided by IBM Corp., as well as the R statistical computing language, version 4.2.2, originating from Vienna, Austria. The URL for R is: http://www.R-project.org/. Numerical variables were presented using the median and interquartile range, while categorical variables were expressed as percentages with their corresponding numbers in parentheses. Statistical analysis was made by Fisher exact test and chi-square test. The Kaplan-Meier method was utilized for computing the survival curves of both DFS and OS, followed by a comparison using the log-rank test. The Cox proportional hazards regression model was employed to identify the underlying independent variables that were associated with DFS and OS. In the multivariate analyses, the hazard ratio (HR) along with the corresponding 95% confidence interval (CI) were calculated for each factor involved. The Nomogram models were additionally developed to assess the DFS and OS rates. The clinical utility of the prediction models was analyzed by using calibration curve and decision curve analysis.

## Results

### Construction and evaluation of GRIm score with survival

**Figure 1:**
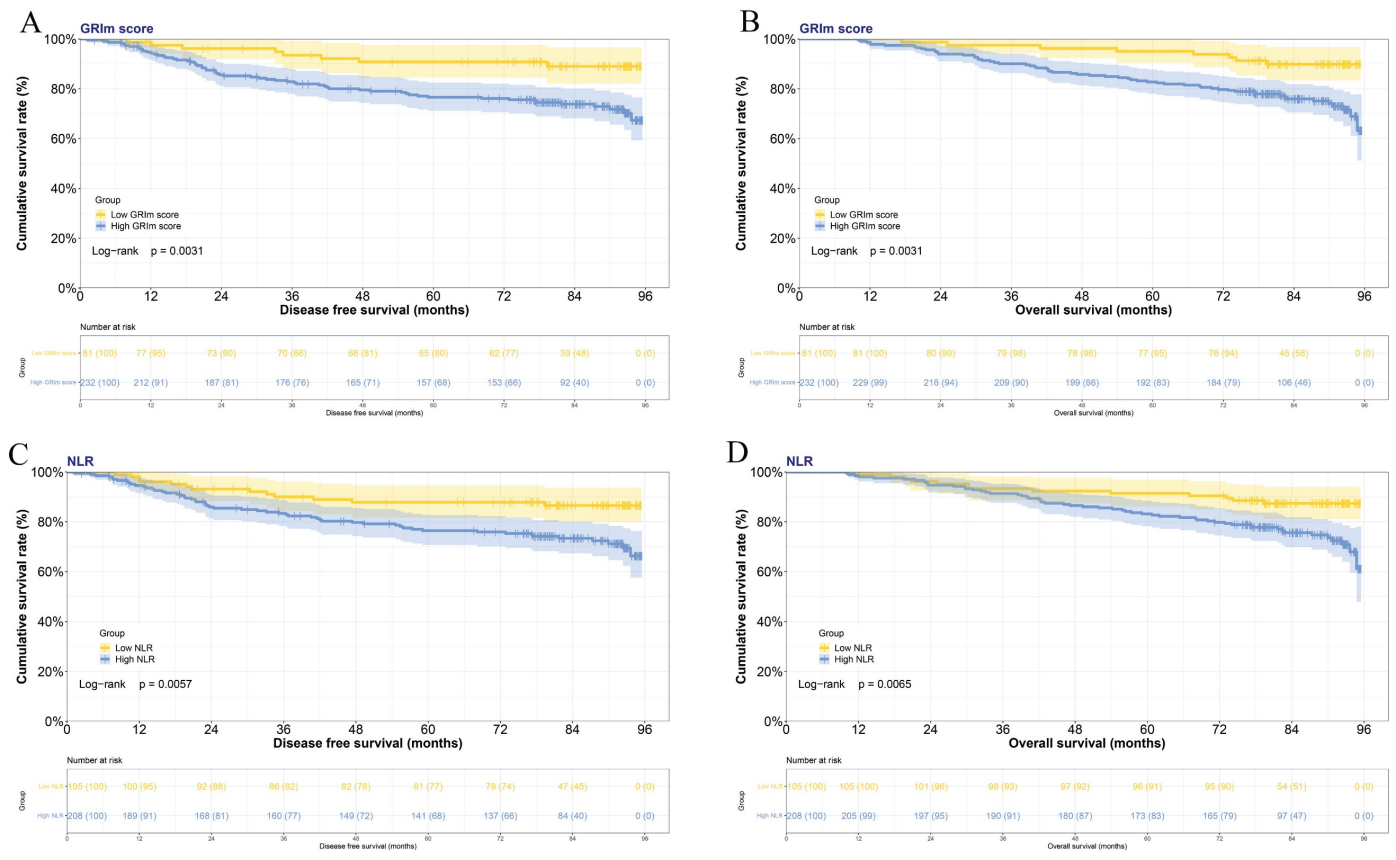
The Kaplan-Meier survival curves illustrating the DFS and the OS among patients with breast cancer, stratified based on the GRIm score or NLR. A) Kaplan-Meier survival curves illustrating the DFS among patients with breast cancer stratified based on the GRIm score; B) Kaplan-Meier survival curves illustrating the OS among patients with breast cancer stratified based on the GRIm score; C) Kaplan-Meier survival curves illustrating the DFS among patients with breast cancer stratified based on the NLR; D) Kaplan-Meier survival curves illustrating the OS among patients with breast cancer stratified based on the NLR.

In this study, the GRIm score was constructed by the albumin level, LDH level, total bilirubin level, AST-to-ALT ratio, and NLR. Based on GRIm scores, there were 81 cases for GRIm score 0 divided into the low-score group, and 232 cases for GRIm score from 1 to 5 points divided into the high-score group. In the low GRIm score group, the mean duration of DFS was 74.39 months, while the mean OS was 83.71 months. Conversely, in the high GRIm score group, the mean DFS was 66.20 months, and the mean OS was 76.40 months. The Kaplan-Meier estimations of both DFS and OS, categorized according to prognostic risk for each GRIm score, are displayed in **Figures 1A and 1B**, respectively. Significant variations in DFS and OS were observed among the different prognostic risk groups for the GRIm score. (DFS: χ^2^=8.729, P=0.0031; OS: χ^2^=8.729, P=0.0031). Moreover, according to the NLR, 105 cases were in the low NLR group, 208 cases were in the high NLR group. In the low NLR group, the mean duration of DFS was 72.05 months, while the mean OS was 81.07 months. By contrast, in the high NLR group, the mean DFS was 66.44 months, and the mean OS was 76.88 months. The Kaplan-Meier estimations of both DFS and OS, categorized according to prognostic risk for NLR, are displayed in **Figures 1C and 1D**, respectively. Significant variations in DFS and OS were observed among the different prognostic risk groups for the NLR (DFS: χ^2^=7.628, P=0.0057; OS: χ^2^=7.416, P=0.0065).

### Baseline characteristics according to the GRIm score

The 313 cases of breast cancer patients were consecutively enrolled in this discovery cohort between January 2015 and November 2015. All enrolled patients were females, the median age was 51 years, with a range of 25 to 78 years. According to the TNM stage, 85 (27.2%) cases were the stage I, 138 (44.1%) cases were the stage II, 90 (28.8%) cases were the stage III. Breast cancer was divided into four distinct molecular subtypes, dependent on the expression profiles of estrogen receptor (ER), progesterone receptor (PR), human epidermal growth factor receptor 2 (HER2), and Ki67. In the study, the distribution of breast cancer molecular subtypes was as follows: 58 cases (18.5%) belonged to the Luminal A subtype, 65 cases (20.8%) were identified as the HER2-enriched subtype, 63 cases (20.1%) were categorized as the Luminal B HER2-negative subtype, another 63 cases (20.1%) fell into the Luminal B HER2-positive subtype, and finally, 64 cases (20.4%) were designated as the Triple-negative subtype. The GRIm score exhibited a statistically significant association with menarche age (P=0.004). Detailed information is shown in **Table 1**.

### Comparison of performance of GRIm score according to common hematological parameters

In this study, we enrolled the common hematological parameters, including AST, ALT, LDH, γ-glutamyl transpeptidase (GGT), albumin (ALB), direct bilirubin (DBIL), indirect bilirubin (IBIL), total bilirubin (TBIL), total protein (TP), globularproteins (G), albumin/globularproteins (A/G), prealbumin (PAB), carcinoembryonic antigen (CEA), cancer antigen 153 (CA153), fibrinogen (FBG), international normalized ratio (INR), D-Dimer (D-D), white blood cell (W), lymphocyte (L), monocyte (M), neutrophil (N), platelet (P). The median values of these enrolled hematological parameters served as the basis for grouping. The GRIm score exhibited a statistically significant association with AST/ALT (P=0.002), FBG (P=0.041), N (P<0.001), L (P<0.001), W (P=0.002), M (P=0.03), and NLR (P<0.001). Detailed information is presented in **Table 2**.

### Comparison of performance of GRIm score according to pathological features

All patients included in this study underwent surgical treatment. Of all enrolled patients, the maximum short diameter of the tumor in 154 (49.2%) patients was less than 2 cm, in 149 (47.6%) patients was between 2 cm and 5cm, in 10 (3.2%) patients was more than 5 cm. The GRIm score exhibited a statistically significant association with positive axillary lymph nodes (PALN) (P=0.042). Details are shown in **Table 3**.

### Correlation between GRIm score and organ metastasis

All patients were followed up after surgery. In the current study, 32 (10.2%) patients had lung metastasis of breast cancer after operation, 40 (12.8%) patients had bone metastasis of breast cancer after operation, 29 (9.3%) patients had liver metastasis of breast cancer after operation, 12 (3.8%) patients had chest wall metastasis of breast cancer after operation, 14 (4.5%) patients had mediastinal metastasis of breast cancer after operation, 16 (5.1%) patients had brain metastasis of breast cancer after operation, 11 (3.5%) patients had pleural metastasis of breast cancer after operation, 159 (50.8%) patients had axillary metastasis of breast cancer after operation, 49 (15.7%) patients had clavicle metastasis of breast cancer after operation, respectively. The GRIm score exhibited a statistically significant association with axillary metastasis (P=0.048). Detailed information is shown in **Table S1**.

### The univariate analysis and multivariate analysis

In the current study, we enrolled the GRIm score, NLR, age, BMI, family history, basic disease, menarche age, menopause, ALT, AST, AST/ALT, LDH, ALB, TBIL, CA153, CEA, D-D, FBG, white blood cell, neutrophil, lymphocyte, monocyte, platelet, tumor size, pathological TNM stage, TALN, PALN, molecular subtype, chemotherapy, radiotherapy, endocrine therapy, targeted therapy to construct the univariate and multivariate COX analysis. For these enrolled variables, we adjusted for the confounding factors, including age, BMI, menarche age. In the univariate analysis, GRIm score, NLR, family history, basic disease, menopause, pathological TNM stage, PALN, chemotherapy, and endocrine therapy were identified as meaningful factors. The multivariate analysis identified the GRIm score, NLR, family history, menopause status, pathological TNM stage, chemotherapy, and endocrine therapy as potential prognostic factors for DFS in **Table 4**. Furthermore, the univariate analysis identified the GRIm score, NLR, family history, ALT, pathological TNM stage, chemotherapy, and endocrine therapy as significant factors. Additionally, the multivariate analysis revealed that the GRIm score, NLR, family history, ALT, pathological TNM stage, chemotherapy, and endocrine therapy were potential predictors of outcome for OS in **Table 5**. For these results, the multivariate analyses demonstrated that the GRIm score significantly and adversely impacted both DFS and OS. (DFS, HR: 2.789, 95% CI: 1.304-5.965, P=0.008; OS: HR: 3.015, 95% CI: 1.409-10.087, P=0.004). After adjusting for variables such as age, BMI, menarche age, the GRIm score was significantly associated with chemotherapy (DFS, HR: 0.214, 95% CI: 0.107-0.427, P<0.001; OS: HR: 0.141, 95% CI: 0.069-0.289, P<0.001) and endocrine therapy (DFS, HR: 0.241, 95% CI: 0.137-0.423, P<0.001; OS: HR: 0.264, 95% CI: 0.148-0.468, P<0.001).

### Nomograms constructed and validated

**Figure 2:**
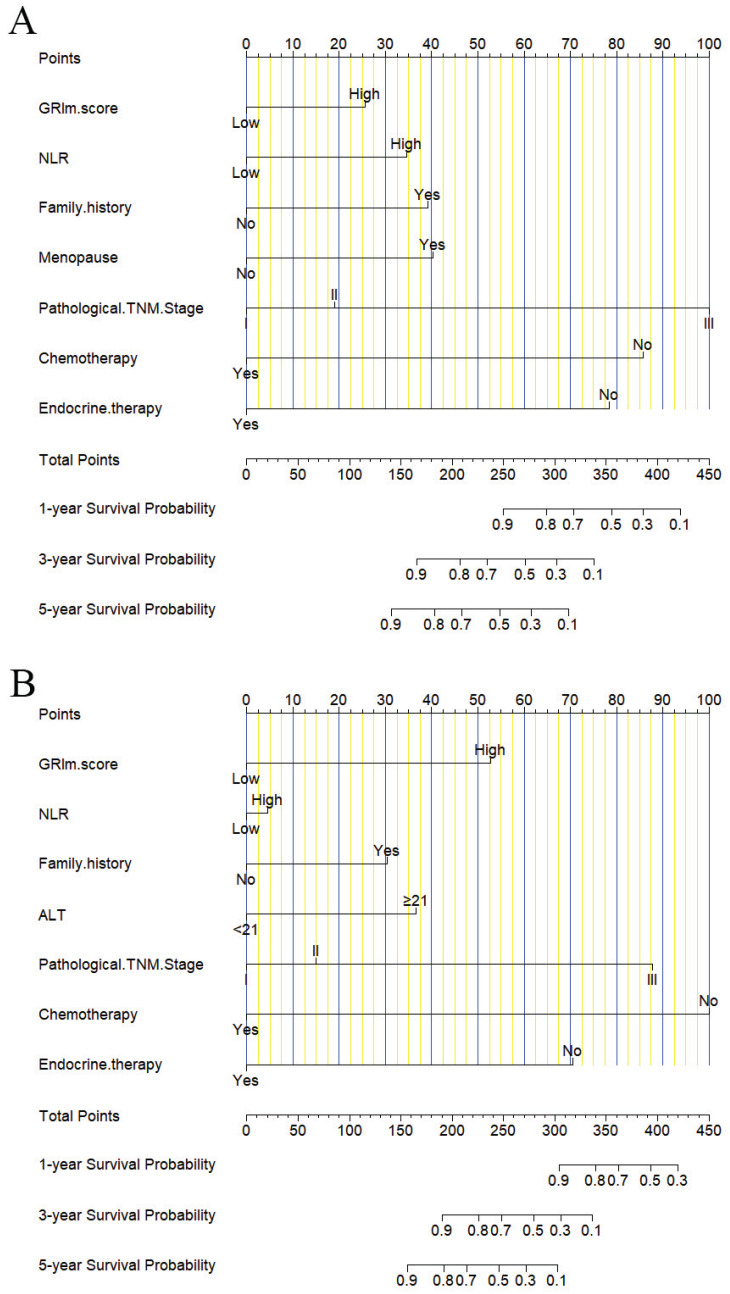
Nomograms to predict the survival time among patients with breast cancer. A) Nomograms to predict the DFS among patients with breast cancer; B) Nomograms to predict the OS among patients with breast cancer.

**Figure 3:**
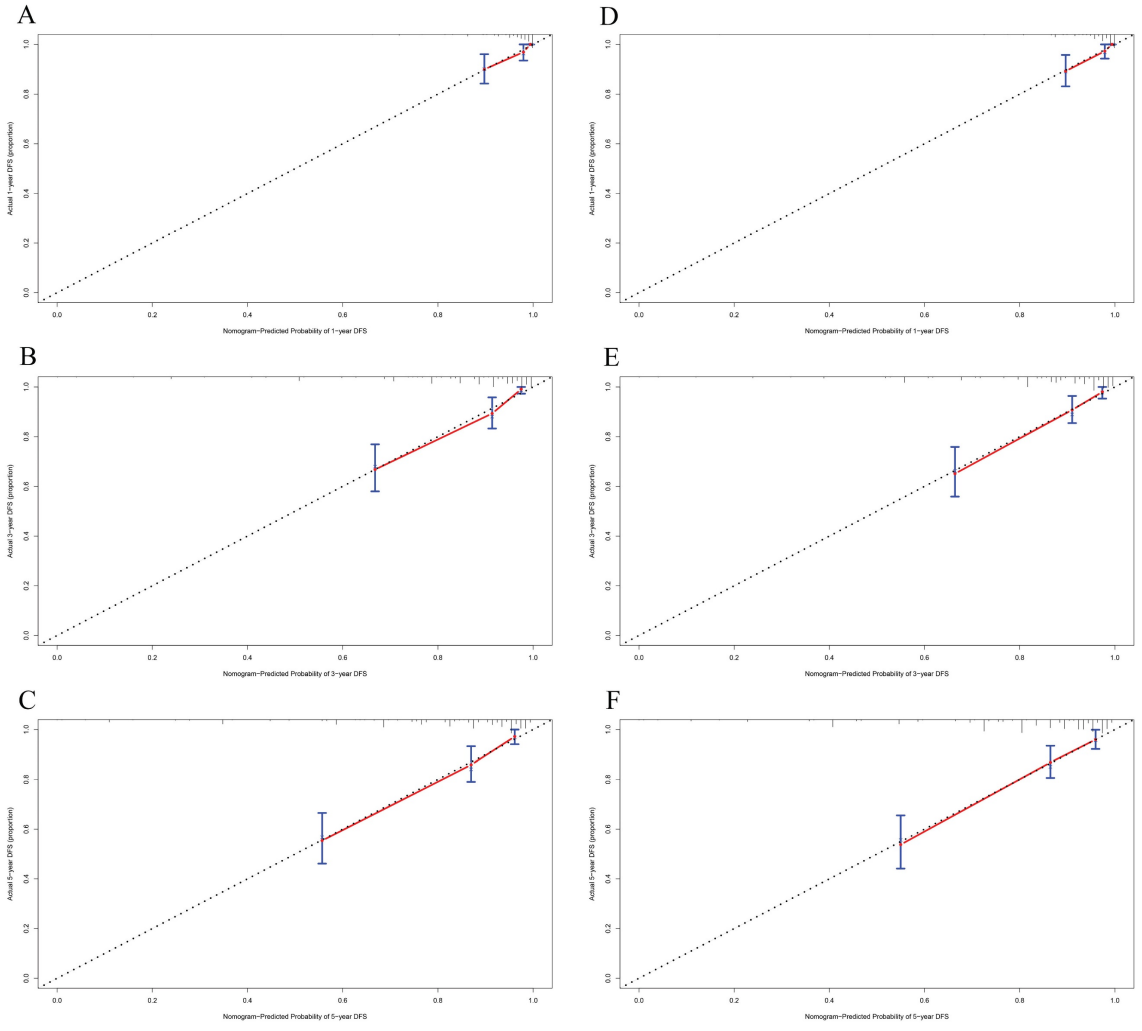
The predicted 1-, 3-, and 5-year DFS rates and OS rates of breast cancer patients using the nomogram closely matched the actual observed values by calibration curves. A) The predicted 1-year DFS rate of breast cancer patients using the nomogram closely matched the actual observed values by calibration curve; B) The predicted 3-year DFS rate of breast cancer patients using the nomogram closely matched the actual observed values by calibration curve; C) The predicted 5-year DFS rate of breast cancer patients using the nomogram closely matched the actual observed values by calibration curve; D) The predicted 1-year OS rate of breast cancer patients using the nomogram closely matched the actual observed values by calibration curve; E) The predicted 3-year OS rate of breast cancer patients using the nomogram closely matched the actual observed values by calibration curve; F) The predicted 5-year OS rate of breast cancer patients using the nomogram closely matched the actual observed values by calibration curve.

**Figure 4:**
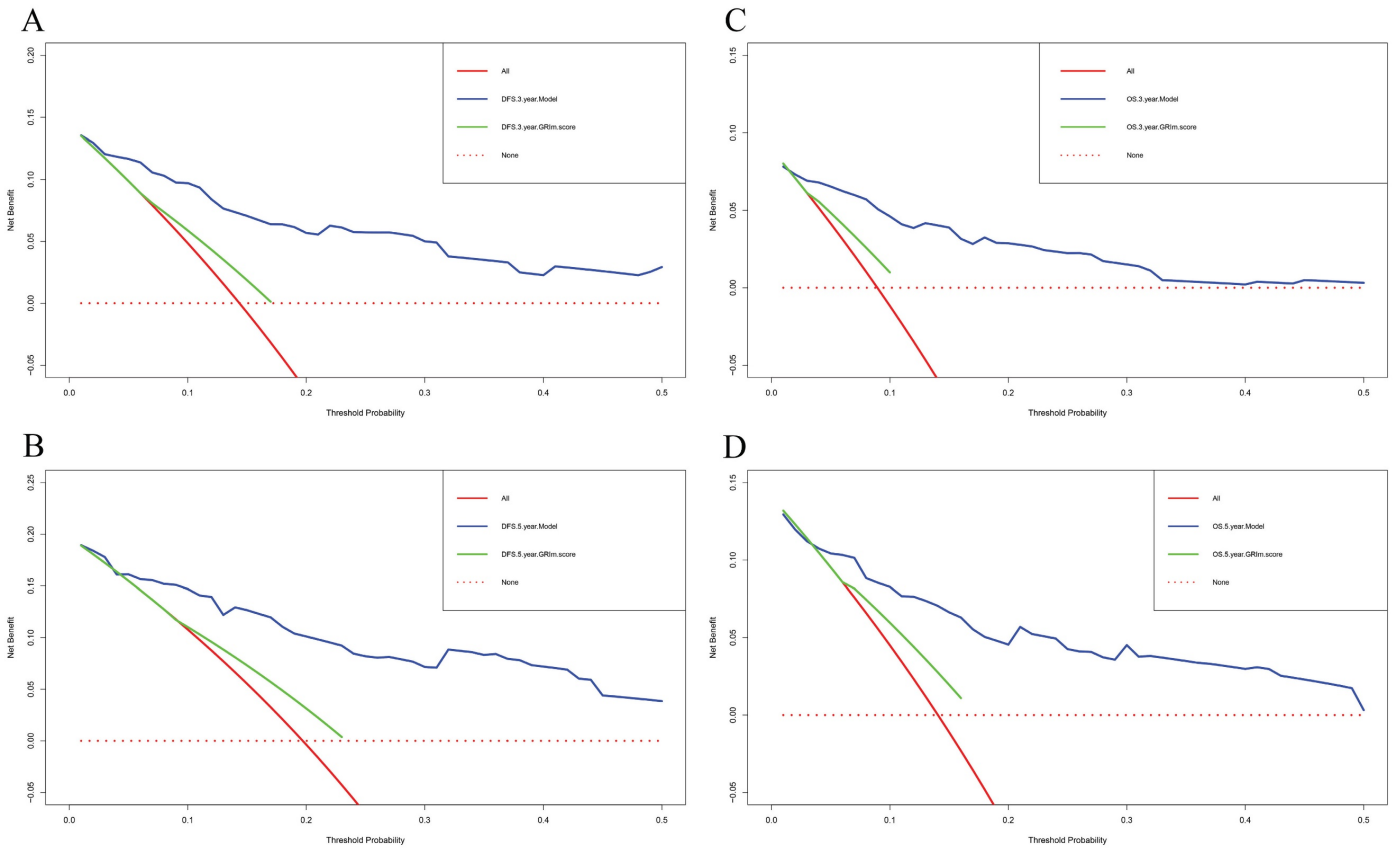
Decision curve analyses (DCA) of nomogram and GRIm score prediction model for predicting the DFS and OS rates in 3- and 5-year. A) DCA of nomogram and GRIm score prediction model for predicting the DFS rates in 3-year; B) DCA of nomogram and GRIm score prediction model for predicting the DFS rates in 5-year; C) DCA of nomogram and GRIm score prediction model for predicting the OS rates in 3-year; D) DCA of nomogram and GRIm score prediction model for predicting the OS rates in 5-year.

In this study, the parameters with P < 0.05, including GRIm score, NLR, family history, menopause, pathological TNM stage, chemotherapy, and endocrine therapy, were selected based on multivariate analyses to construct a nomogram for the prediction of DFS in **Figure 2A**. The C-index for this nomogram model predicting DFS was 0.823 (95%CI: 0.699-0.903). Furthermore, the parameters with P < 0.05, including GRIm score, NLR, family history, ALT, pathological TNM stage, chemotherapy, and endocrine therapy by multivariate analyses were chose to comprise nomogram for the prediction of OS in **Figure 2B**. The C-index for this nomogram model predicting OS was 0.807 (95%CI: 0.684-0.890). The calibration curves used to predict DFS at 1-, 3-, and 5-year intervals exhibited significant correlation between predictions and actual observations, indicating minimal departure from an ideal fit in **Figure 3A-C**. Also, the calibration curves used to evaluate 1-, 3-, and 5-year OS showed that good correlation between predictions and actual observations, and no significant deviation from perfect fit in **Figure 3D-F**. Furthermore, the nomograms constructed indicated a superior positive net benefit compared to the GRIm score in predicting 3- and 5-year DFS, as shown in **Figure 4A-B**, and in predicting 3- and 5-year DFS in **Figure 4C-D**. In addition, the nomograms constructed indicated a better positive net benefit than NLR in predicting 3- and 5-year DFS, as illustrated in **Figure S1A-B**, and in predicting 3- and 5-year DFS in **Figure S1C-D**.

### Subgroup analyses

According to multivariate analyses, TNM stage was also a potential factor in this study. Of those patients, 85 (27.2%) cases were stage I breast cancer, 138 (44.1%) cases were stage II breast cancer, 90 (28.8%) cases were stage III breast cancer, respectively. The mean DFS and OS were 75.27 and 83.17 months in stage I breast cancer, 73.58 and 83.05 months in stage II breast cancer, 53.69 and 68.58 months in stage III breast cancer, respectively. Kaplan-Meier estimations of both DFS and OS, categorized according to prognostic risk for each TNM stage, are presented in **Figure S2A and S2B**. Significant variations in both DFS and OS were observed among the distinct TNM stage groups. (DFS: χ^2^=30.140, P<0.0001; OS: χ^2^=260.43, P<0.0001). We analyzed the relationship between TNM stage and the common hematological parameters. Compared to patients with stage I or stage II, patients with stage III were notably linked to TBIL (P=0.002), CA153 (P=0.001), CEA (P=0.004), monocyte (P=0.031). Detailed information is shown in **Table S2**. Furthermore, we also analyzed the prognostic effect of the GRIm score and its components by different TNM stage. The results indicated that the GRIm score was related to NLR, neutrophil, and lymphocyte. Detailed information is shown in **Table S3**.

Based on multivariate analyses, chemotherapy and endocrine therapy were also a potential factor in this study. We further analyzed the data and found that patients who received chemotherapy had longer survival time than those who did not receive chemotherapy (DFS: P=0.00084; OS: P=0.00034). Moreover, for those received chemotherapy patients (290 cases), patients with low GRIm score group had survived longer than those with high GRIm score group (DFS: P=0.0073; OS: P=0.0075). We also analyzed the effects of endocrine therapy and found that patients who received endocrine therapy had longer survival times than those who did not receive it (DFS: P<0.0001; OS: P<0.0001). Moreover, for those who received endocrine therapy patients (163 cases), patients with low GRIm score group had survived longer than those with high GRIm score group (DFS: P=0.026; OS: P=0.030).

## Discussion

In this study, the GRIm score was constructed by the albumin level, LDH level, total bilirubin level, AST-to-ALT ratio, and NLR. To our knowledge, the GRIm score serves as a valuable predictor of survival outcomes among patients with diverse malignant tumor types, such as esophageal squamous cell carcinoma 15, non-small cell lung cancer 16, hepatocellular carcinoma 17, operable pancreatic adenocarcinoma 18. Despite its potential utility, limited studies have explored the predictive value of the GRIm score specifically in the context of breast cancer. Our study concluded that the GRIm score serves as an independent predictor of DFS and OS in patients with breast cancer. Our findings revealed that patients exhibiting a low GRIm score exhibited prolonged DFS and OS.

The blood albumin level, a widely recognized nutritional index, has been established to have a correlation with liver function and prognosis. In Tanriverdi O's study, they demonstrated that patients with low serum albumin levels exhibited shorter PFS and OS. Furthermore, a reduced level of serum albumin was determined as a standalone factor that was significantly linked to a worse outcome in patients with Stage IIIB non-small cell lung cancer 19. Fujii T's study investigated the relationship between serum levels of albumin and breast cancer, and demonstrated that low serum albumin levels were related to worse prognosis, but not a stand-alone prognostic indicator for the tumor microenvironment in breast cancer 20.

As is known to all, the serum level of total bilirubin reflects the liver function. Serum bilirubin is the final product of blood metabolism and has many protective properties, such as anti-inflammatory, antioxidant, and anticancer activities; and it is negatively correlated with various malignant tumors 21. In Gao C's study, their results indicated that higher serum direct bilirubin concentrations were related to the increased risk of poor prognosis in rectal cancer 22. Another study showed that the serum total bilirubin levels prior to treatment may serve as a potential biomarker for anticipating the clinical outcomes among patients diagnosed with primary central nervous system lymphoma undergoing a combination of chemotherapy and immunochemotherapy 23. According to these studies, the results were summarized as follows those patients with lower albumin levels or higher total bilirubin levels had poor survival.

Lactate dehydrogenase (LDH) serves as a well-recognized biomarker of inflammatory responses, and is detected in many tissues of the human body. LDH not only is a simple indicator of tumor burden but also is a complex biomarker associated with immunogenicity, metabolic activity, and invasiveness of numerous tumors 24. A meta-analysis examined the association between LDH levels prior to treatment and clinical results in patients with non-small cell lung cancer, indicating that patients with elevated LDH values lead to poorer PFS and OS 25.

The levels of AST and ALT are widely recognized markers for assessing the preservation of liver function. A prior study indicated that the serum AST-to-ALT ratio emerged as a reliable prognostic factor for DFS, but not for OS in patients suffering from colorectal cancer that was non-metastatic and at stages II and III 26. Wang F's study indicated that the pre-surgical serum AST-to-ALT ratio might serve as a predictive marker for patients with hepatocellular carcinoma undergoing simultaneous thermal ablation and transarterial chemoembolization 27. Another study also indicated that a higher ratio of AST-to-ALT in the blood serum was associated with a poorer prognosis for patients with HBV-induced hepatocellular carcinoma receiving hepatectomy 28. Even though the precise mechanism is not yet clear, a speculated mechanism is that as liver function becomes increasingly impaired, invasive tumor progression results in a marked elevation of AST levels and a concomitant reduction in the rate of AST clearance 28. Therefore, we assume that an elevated AST-to-ALT ratio can be applied to impair liver function and predict the prognosis of tumors.

Inflammation, being a vital part of the tumor microenvironment, is instrumental in driving cancer progression 29. As a sensitive biomarker of inflammation, NLR has different effects on the development of tumors 30. Certain studies have shown that tumor-stimulated neutrophils facilitate angiogenesis, immune suppression, and enhance the infiltration, migration, and metastatic abilities of tumor cells 31. In contrast, the number of tumor-infiltrating lymphocytes can vary among different types of tumors, and its quantitation has been explored as a potential means to enhance clinical response to chemotherapy. Exactly, the increased neutrophil count often indicates a higher risk of inflammation, while a higher lymphocyte count signifies a stronger immune response. Therefore, we hypothesize that an elevated NLR can potentially be used to predict the prognosis of tumors, as it reflects the balance between inflammation and immune response within the tumor microenvironment.

According to the above biological mechanisms, it is entirely plausible to anticipate expected cancer patient survival rates with GRIm score. The relationship between GRIm score and the prognosis of malignant tumors has been determined 18,32,33. The current study suggests that patients with a high GRIm score exhibited a poorer prognosis and shorter survival time (DFS, HR: 2.789, 95% CI: 1.304-5.965, P=0.008; OS: HR: 3.015, 95% CI: 1.409-10.087, P=0.004) in early breast cancer. The utility of neutrophils and lymphocytes in tumors and immunotherapy is reported. NLR was one of the comprised of GRIm score factors. Then, the patients in high NLR value had poor prognosis and short survival time (DFS, HR: 2.500, 95% CI: 1.349-4.636, P=0.004; OS: HR: 2.225, 95% CI: 1.203-4.116, P=0.011). Moreover, the multivariate analysis identified the GRIm score, NLR, TNM stage, chemotherapy, and endocrine therapy as potential prognostic factors. TNM stage was the common factor for predicting prognosis for tumors. The GRIm score was related to NLR, neutrophil, and lymphocyte by different TNM stage. Moreover, patients, who received chemotherapy or endocrine therapy, with low GRIm score group had survived longer than those with high GRIm score group. Furthermore, nomograms were constructed by these prognostic factors. In addition, the nomograms constructed by GRIm score performed superior predictive capabilities than NLR or only by GRIm score.

It's worth noting that the present research has various constraints that ought to be considered. Firstly, our retrospective analysis was restricted to data from one single institution only, thus our findings necessitate further prospective validation through multicenter studies involving independent patient groups. Secondly, due to the restricted duration of observation in the present study, a more extended period of observation could potentially have led to different findings. Lastly, the nomograms constructed need to be tested and verified by an external validation cohort in the following study.

## Conclusion

In conclusion, our pooled results demonstrate that GRIm score, as a novel prognostic score, could be a valuable prognostic tool for breast cancer patients. The nomograms formulated using the GRIm score have demonstrated their capacity to precisely forecast the DFS and OS rates among breast cancer patients. The GRIm score can help oncologists discuss prognosis, treatment decisions, and patient selection for clinical trials.

## Supplementary Material

Supplementary figures and tables.

## Figures and Tables

**Table 1:** Baseline characteristics according to the GRIm score.

Parameters	level	Overall	Low GRIm score	High GRIm score	p
n		313	81	232	
Age	<51	154 (49.2)	39 (48.1)	115 (49.6)	0.927
	≥51	159 (50.8)	42 (51.9)	117 (50.4)	
Weight	<62	149 (47.6)	37 (45.7)	112 (48.3)	0.784
	≥62	164 (52.4)	44 (54.3)	120 (51.7)	
Height	<1.60	105 (33.5)	22 (27.2)	83 (35.8)	0.202
	≥1.60	208 (66.5)	59 (72.8)	149 (64.2)	
BMI	<23.8	156 (49.8)	37 (45.7)	119 (51.3)	0.459
	≥23.8	157 (50.2)	44 (54.3)	113 (48.7)	
Family history	No	242 (77.3)	60 (74.1)	182 (78.4)	0.512
	Yes	71 (22.7)	21 (25.9)	50 (21.6)	
Basic disease	No	244 (78.0)	66 (81.5)	178 (76.7)	0.463
	Yes	69 (22.0)	15 (18.5)	54 (23.3)	
Hypertension	No	273 (87.2)	73 (90.1)	200 (86.2)	0.474
	Yes	40 (12.8)	8 (9.9)	32 (13.8)	
Diabetes mellitus	No	297 (94.9)	77 (95.1)	220 (94.8)	1.000
	Yes	16 (5.1)	4 (4.9)	12 (5.2)	
Coronary heart disease	No	300 (95.8)	77 (95.1)	223 (96.1)	0.930
	Yes	13 (4.2)	4 (4.9)	9 (3.9)	
Menarche age	<15	121 (38.7)	20 (24.7)	101 (43.5)	0.004
	≥15	192 (61.3)	61 (75.3)	131 (56.5)	
Menopause	No	152 (48.6)	33 (40.7)	119 (51.3)	0.132
	Yes	161 (51.4)	48 (59.3)	113 (48.7)	
Primary tumor site	Upper outer quadrant	178 (56.9)	44 (54.3)	134 (57.8)	0.359
	Lower outer quadrant	29 (9.3)	10 (12.3)	19 (8.2)	
	Lower inner quadrant	23 (7.3)	3 (3.7)	20 (8.6)	
	Upper inner quadrant	44 (14.1)	11 (13.6)	33 (14.2)	
	Central	39 (12.5)	13 (16.0)	26 (11.2)	
Operative time	<75	143 (45.7)	30 (37.0)	113 (48.7)	0.092
	≥75	170 (54.3)	51 (63.0)	119 (51.3)	
Type of surgery	Mastectomy	293 (93.6)	74 (91.4)	219 (94.4)	0.485
	Breast-conserving surgery	20 (6.4)	7 (8.6)	13 (5.6)	
Pathological T Stage	T1	167 (53.4)	45 (55.6)	122 (52.6)	0.802
	T2	134 (42.8)	33 (40.7)	101 (43.5)	
	T3	10 (3.2)	2 (2.5)	8 (3.4)	
	T4	2 (0.6)	1 (1.2)	1 (0.4)	
Pathological N Stage	N0	129 (41.2)	38 (46.9)	91 (39.2)	0.377
	N1	97 (31.0)	26 (32.1)	71 (30.6)	
	N2	50 (16.0)	11 (13.6)	39 (16.8)	
	N3	37 (11.8)	6 (7.4)	31 (13.4)	
Pathological TNM Stage	I	85 (27.2)	25 (30.9)	60 (25.9)	0.305
	II	138 (44.1)	38 (46.9)	100 (43.1)	
	III	90 (28.8)	18 (22.2)	72 (31.0)	
Molecular subtype	Luminal A	58 (18.5)	18 (22.2)	40 (17.2)	0.712
	Luminal B HER2+	63 (20.1)	17 (21.0)	46 (19.8)	
	Luminal B HER2-	63 (20.1)	15 (18.5)	48 (20.7)	
	HER2 enriched	65 (20.8)	18 (22.2)	47 (20.3)	
	Triple negative	64 (20.4)	13 (16.0)	51 (22.0)	
Chemotherapy	No	23 (7.3)	10 (12.3)	13 (5.6)	0.079
	Yes	290 (92.7)	71 (87.7)	219 (94.4)	
Radiotherapy	No	220 (70.3)	53 (65.4)	167 (72.0)	0.332
	Yes	93 (29.7)	28 (34.6)	65 (28.0)	
Endocrine therapy	No	150 (47.9)	35 (43.2)	115 (49.6)	0.391
	Yes	163 (52.1)	46 (56.8)	117 (50.4)	
Targeted therapy	No	279 (89.1)	69 (85.2)	210 (90.5)	0.263
	Yes	34 (10.9)	12 (14.8)	22 (9.5)	

Abbreviation: GRIm score: Gustave Roussy Immune score; BMI: body mass index.

**Table 2:** A performance comparison between GRIm score and common hematological parameters.

Parameters	level	Overall	Low GRIm score	High GRIm score	p
n		313	81	232	
ALT	<21	142 (45.4)	33 (40.7)	109 (47.0)	0.400
	≥21	171 (54.6)	48 (59.3)	123 (53.0)	
AST	<23	147 (47.0)	36 (44.4)	111 (47.8)	0.690
	≥23	166 (53.0)	45 (55.6)	121 (52.2)	
AST/ALT	<1.44	267 (85.3)	78 (96.3)	189 (81.5)	0.002
	≥1.44	46 (14.7)	3 (3.7)	43 (18.5)	
LDH	<170	156 (49.8)	38 (46.9)	118 (50.9)	0.629
	≥170	157 (50.2)	43 (53.1)	114 (49.1)	
GGT	<14	142 (45.4)	41 (50.6)	101 (43.5)	0.331
	≥14	171 (54.6)	40 (49.4)	131 (56.5)	
ALB	<45	145 (46.3)	43 (53.1)	102 (44.0)	0.198
	≥45	168 (53.7)	38 (46.9)	130 (56.0)	
TBIL	<12.45	156 (49.8)	44 (54.3)	112 (48.3)	0.419
	≥12.45	157 (50.2)	37 (45.7)	120 (51.7)	
DBIL	<3.9	155 (49.5)	47 (58.0)	108 (46.6)	0.099
	≥3.9	158 (50.5)	34 (42.0)	124 (53.4)	
IBIL	<8.29	156 (49.8)	43 (53.1)	113 (48.7)	0.583
	≥8.29	157 (50.2)	38 (46.9)	119 (51.3)	
TP	<74	132 (42.2)	33 (40.7)	99 (42.7)	0.863
	≥74	181 (57.8)	48 (59.3)	133 (57.3)	
G	<29	137 (43.8)	31 (38.3)	106 (45.7)	0.304
	≥29	176 (56.2)	50 (61.7)	126 (54.3)	
A/G	<1.5	104 (33.2)	34 (42.0)	70 (30.2)	0.071
	≥1.5	209 (66.8)	47 (58.0)	162 (69.8)	
PAB	<267	156 (49.8)	36 (44.4)	120 (51.7)	0.318
	≥267	157 (50.2)	45 (55.6)	112 (48.3)	
CA153	<9.82	156 (49.8)	44 (54.3)	112 (48.3)	0.419
	≥9.82	157 (50.2)	37 (45.7)	120 (51.7)	
CEA	<1.49	156 (49.8)	35 (43.2)	121 (52.2)	0.209
	≥1.49	157 (50.2)	46 (56.8)	111 (47.8)	
D-D	<0.25	151 (48.2)	41 (50.6)	110 (47.4)	0.713
	≥0.25	162 (51.8)	40 (49.4)	122 (52.6)	
FBG	<2.6	153 (48.9)	48 (59.3)	105 (45.3)	0.041
	≥2.6	160 (51.1)	33 (40.7)	127 (54.7)	
INR	<0.97	139 (44.4)	43 (53.1)	96 (41.4)	0.090
	≥0.97	174 (55.6)	38 (46.9)	136 (58.6)	
White blood cell	<5.45	156 (49.8)	53 (65.4)	103 (44.4)	0.002
	≥5.45	157 (50.2)	28 (34.6)	129 (55.6)	
Neutrophil	<3.23	155 (49.5)	68 (84.0)	87 (37.5)	<0.001
	≥3.23	158 (50.5)	13 (16.0)	145 (62.5)	
Lymphocyte	<1.70	154 (49.2)	19 (23.5)	135 (58.2)	<0.001
	≥1.70	159 (50.8)	62 (76.5)	97 (41.8)	
NLR	Low	105 (33.5)	80 (98.8)	25 (10.8)	<0.001
	High	208 (66.5)	1 (1.2)	207 (89.2)	
Monocyte	<0.35	151 (48.2)	48 (59.3)	103 (44.4)	0.030
	≥0.35	162 (51.8)	33 (40.7)	129 (55.6)	
Platelet	<233	153 (48.9)	37 (45.7)	116 (50.0)	0.589
	≥233	160 (51.1)	44 (54.3)	116 (50.0)	

Abbreviation: GRIm score: Gustave Roussy Immune score; ALT: alanine aminotransferase; AST: aspartate aminotransferase; LDH: lactate dehydrogenase; GGT: γ-glutamyl transpeptidase; ALB: albumin; TBIL: total bilirubin; DBIL: direct bilirubin; IBIL: indirect bilirubin; TP: total protein; G: globularproteins; PAB: prealbumin; CA153: cancer antigen 153; CEA: carcinoembryonic antigen; D-D: D-Dimer; FBG: fibrinogen; INR: international normalized ratio; NLR: neutrophil to lymphocyte ratio.

**Table 3:** A performance comparison between GRIm score and pathological features.

Parameters	level	Overall	Low GRIm score	High GRIm score	p
n		313	81	232	
Tumor size	≤2	154 (49.2)	43 (53.1)	111 (47.8)	0.420
	>2 and<5	149 (47.6)	37 (45.7)	112 (48.3)	
	≥5	10 (3.2)	1 (1.2)	9 (3.9)	
Total lymph nodes (TLN)	<16	149 (47.6)	40 (49.4)	109 (47.0)	0.808
	≥16	164 (52.4)	41 (50.6)	123 (53.0)	
Positive lymph nodes (PLN)	<1	134 (42.8)	39 (48.1)	95 (40.9)	0.319
	≥1	179 (57.2)	42 (51.9)	137 (59.1)	
Total axillary lymph nodes (TALN)	<14	149 (47.6)	42 (51.9)	107 (46.1)	0.447
	≥14	164 (52.4)	39 (48.1)	125 (53.9)	
Positive axillary lymph nodes (PALN)	<1	157 (50.2)	49 (60.5)	108 (46.6)	0.042
	≥1	156 (49.8)	32 (39.5)	124 (53.4)	
ER	0-25%	144 (46.0)	36 (44.4)	108 (46.6)	0.918
	26-50%	26 (8.3)	6 (7.4)	20 (8.6)	
	51-75%	48 (15.3)	12 (14.8)	36 (15.5)	
	76-100%	95 (30.4)	27 (33.3)	68 (29.3)	
PR	0-25%	192 (61.3)	51 (63.0)	141 (60.8)	0.709
	26-50%	35 (11.2)	7 (8.6)	28 (12.1)	
	51-75%	35 (11.2)	11 (13.6)	24 (10.3)	
	76-100%	51 (16.3)	12 (14.8)	39 (16.8)	
HER2	Negative	185 (59.1)	46 (56.8)	139 (59.9)	0.718
	Positive	128 (40.9)	35 (43.2)	93 (40.1)	
Ki67	0-25%	147 (47.0)	42 (51.9)	105 (45.3)	0.568
	26-50%	105 (33.5)	25 (30.9)	80 (34.5)	
	51-75%	46 (14.7)	12 (14.8)	34 (14.7)	
	76-100%	15 (4.8)	2 (2.5)	13 (5.6)	
CK5/6	Negative	221 (70.6)	57 (70.4)	164 (70.7)	1.000
	Positive	92 (29.4)	24 (29.6)	68 (29.3)	
E-cad	Negative	11 (3.5)	5 (6.2)	6 (2.6)	0.247
	Positive	302 (96.5)	76 (93.8)	226 (97.4)	
P120	Negative	293 (93.6)	76 (93.8)	217 (93.5)	1.000
	Positive	20 (6.4)	5 (6.2)	15 (6.5)	
P53	Negative	170 (54.3)	43 (53.1)	127 (54.7)	0.898
	Positive	143 (45.7)	38 (46.9)	105 (45.3)	
Blood vessel invasion	No	289 (92.3)	76 (93.8)	213 (91.8)	0.730
	Yes	24 (7.7)	5 (6.2)	19 (8.2)	

Abbreviation: GRIm score: Gustave Roussy Immune score; ER: estrogen receptor; PR: progesterone receptor; HER2: human epidermal growth factor receptor 2; E-cad: E-cadherin.

**Table 4:** The univariate analysis and multivariate analysis for DFS.

Parameters	Group		Univariate				Multivariate		
		P	HR	95% CI		P	HR	95% CI	
				Low	High			Low	High
GRIm score	Low	0.005	1(Ref.)			0.008	1(Ref.)		
	High		2.891	1.382	6.047		2.789	1.304	5.965
NLR	Low	0.007	1(Ref.)			0.004	1(Ref.)		
	High		2.288	1.250	4.188		2.500	1.349	4.636
Age*	<51	0.743	1(Ref.)						
	≥51		0.860	0.350	2.113				
BMI*	<23.8	0.534	1(Ref.)						
	≥23.8		0.819	0.437	1.537				
Family history	No	0.008	1(Ref.)			0.015	1(Ref.)		
	Yes		2.435	1.265	4.687		1.964	1.137	3.391
Basic disease	No	0.015	1(Ref.)			0.052	1(Ref.)		
	Yes		2.190	1.165	4.115		1.730	0.995	3.010
Menarche age*	<15	0.131	1(Ref.)						
	≥15		1.618	0.866	3.023				
Menopause	No	0.000	1(Ref.)			0.011	1(Ref.)		
	Yes		2.886	1.698	4.905		2.084	1.183	3.671
ALT	<21	0.080	1(Ref.)						
	≥21		2.327	0.905	5.982				
AST	<23	0.531	1(Ref.)						
	≥23		0.805	0.408	1.588				
AST/ALT	<1.44	0.348	1(Ref.)						
	≥1.44		1.445	0.669	3.121				
LDH	<170	0.850	1(Ref.)						
	≥170		1.063	0.564	2.004				
ALB	<45	0.114	1(Ref.)						
	≥45		0.623	0.347	1.120				
TBIL	<12.45	0.678	1(Ref.)						
	≥12.45		1.139	0.615	2.111				
CA153	<9.82	0.309	1(Ref.)						
	≥9.82		1.373	0.746	2.526				
CEA	<1.49	0.203	1(Ref.)						
	≥1.49		1.514	0.800	2.868				
D-D	<0.25	0.077	1(Ref.)						
	≥0.25		0.594	0.333	1.058				
FBG	<2.6	0.984	1(Ref.)						
	≥2.6		1.007	0.538	1.884				
White blood cell	<5.45	0.836	1(Ref.)						
	≥5.45		0.906	0.356	2.306				
Neutrophil	<3.23	0.970	1(Ref.)						
	≥3.23		0.982	0.375	2.571				
Lymphocyte	<1.70	0.514	1(Ref.)						
	≥1.70		0.853	0.530	1.374				
Monocyte	<0.35	0.266	1(Ref.)						
	≥0.35		0.691	0.360	1.325				
Platelet	<233	0.689	1(Ref.)						
	≥233		0.877	0.460	1.670				
P Tumor size	≤2	0.893	1(Ref.)						
	>2 and<5	0.866	1.062	0.527	2.139				
	≥5	0.726	0.784	0.201	3.057				
Pathological TNM Stage	I	0.000	1(Ref.)			0.000	1(Ref.)		
	II	0.104	2.251	0.847	5.982	0.370	1.401	0.670	2.932
	III	0.000	24.936	5.617	110.705	0.000	6.100	3.005	12.383
TALN	<14	0.674	1(Ref.)						
	≥14		1.108	0.687	1.786				
PALN	<1	0.004	1(Ref.)						
	≥1		2.053	1.253	3.365				
Molecular subtype	Luminal A	0.345	1(Ref.)						
	Luminal B HER2+	0.103	3.258	0.786	13.498				
	Luminal B HER2-	0.091	3.439	0.822	14.378				
	HER2 enriched	0.391	2.064	0.394	10.811				
	Triple negative	0.382	2.059	0.408	10.399				
Chemotherapy	No	0.000	1(Ref.)			0.000	1(Ref.)		
	Yes		0.166	0.065	0.423		0.214	0.107	0.427
Radiotherapy	No	0.456	1(Ref.)						
	Yes		0.736	0.328	1.650				
Endocrine therapy	No	0.000	1(Ref.)			0.000	1(Ref.)		
	Yes		0.178	0.069	0.461		0.241	0.137	0.423
Targeted therapy	No	0.814	1(Ref.)						
	Yes		0.882	0.308	2.521				

*Confounding factor. Abbreviation: DFS: disease free survival; GRIm score: Gustave Roussy Immune score; BMI: body mass index; ALT: alanine aminotransferase; AST: aspartate aminotransferase; LDH: lactate dehydrogenase; GGT: γ-glutamyl transpeptidase; ALB: albumin; TBIL: total bilirubin; DBIL: direct bilirubin; IBIL: indirect bilirubin; TP: total protein; G: globularproteins; PAB: prealbumin; CA153: cancer antigen 153; CEA: carcinoembryonic antigen; D-D: D-Dimer; FBG: fibrinogen; INR: international normalized ratio; NLR: neutrophil to lymphocyte ratio; ER: estrogen receptor; PR: progesterone receptor; HER2: human epidermal growth factor receptor 2; E-cad: E-cadherin.

**Table 5:** The univariate analysis and multivariate analysis for OS.

Parameters	Group		Univariate				Multivariate		
		P	HR	95% CI		P	HR	95% CI	
				Low	High			Low	High
GRIm score	Low	0.005	1(Ref.)			0.004	1(Ref.)		
	High		3.613	1.383	13.708		3.015	1.409	10.087
NLR	Low	0.008	1(Ref.)			0.011	1(Ref.)		
	High		2.264	1.237	4.145		2.225	1.203	4.116
Age*	<51	0.446	1(Ref.)						
	≥51		0.688	0.263	1.800				
BMI*	<23.8	0.629	1(Ref.)						
	≥23.8		0.858	0.460	1.600				
Family history	No	0.027	1(Ref.)			0.011	1(Ref.)		
	Yes		1.772	1.066	2.946		2.020	1.174	3.473
Basic disease	No	0.115	1(Ref.)						
	Yes		1.675	0.882	3.179				
Menarche age*	<15	0.401	1(Ref.)						
	≥15		1.296	0.708	2.370				
Menopause	No	0.052	1(Ref.)						
	Yes		2.732	0.990	7.536				
ALT	<21	0.010	1(Ref.)			0.002	1(Ref.)		
	≥21		3.545	1.350	9.306		2.301	1.357	3.901
AST	<23	0.505	1(Ref.)						
	≥23		0.792	0.399	1.572				
AST/ALT	<1.44	0.453	1(Ref.)						
	≥1.44		1.342	0.622	2.895				
LDH	<170	0.440	1(Ref.)						
	≥170		0.784	0.423	1.454				
ALB	<45	0.478	1(Ref.)						
	≥45		0.842	0.523	1.355				
TBIL	<12.45	0.659	1(Ref.)						
	≥12.45		1.151	0.616	2.150				
CA153	<9.82	0.913	1(Ref.)						
	≥9.82		1.034	0.565	1.895				
CEA	<1.49	0.067	1(Ref.)						
	≥1.49		1.837	0.959	3.517				
D-D	<0.25	0.125	1(Ref.)						
	≥0.25		0.636	0.357	1.133				
FBG	<2.6	0.938	1(Ref.)						
	≥2.6		0.975	0.516	1.843				
White blood cell	<5.45	0.887	1(Ref.)						
	≥5.45		1.072	0.410	2.804				
Neutrophil	<3.23	0.650	1(Ref.)						
	≥3.23		0.806	0.318	2.044				
Lymphocyte	<1.70	0.462	1(Ref.)						
	≥1.70		0.836	0.519	1.347				
Monocyte	<0.35	0.727	1(Ref.)						
	≥0.35		0.890	0.461	1.715				
Platelet	<233	0.807	1(Ref.)						
	≥233		0.924	0.491	1.740				
P Tumor size	≤2	0.964	1(Ref.)						
	>2 and<5	0.790	0.909	0.448	1.842				
	≥5	0.960	0.967	0.259	3.606				
Pathological TNM Stage	I	0.000	1(Ref.)			0.000	1(Ref.)		
	II	0.085	2.482	0.883	6.980	0.393	1.383	0.657	2.907
	III	0.000	24.430	5.325	112.074	0.000	9.852	4.540	21.383
TALN	<14	0.703	1(Ref.)						
	≥14		1.097	0.681	1.768				
PALN	<1	0.284	1(Ref.)						
	≥1		0.554	0.188	1.632				
Molecular subtype	Luminal A	0.256	1(Ref.)						
	Luminal B HER2+	0.114	3.144	0.759	13.021				
	Luminal B HER2-	0.118	3.032	0.756	12.163				
	HER2 enriched	0.322	2.307	0.441	12.079				
	Triple negative	0.669	1.437	0.273	7.569				
Chemotherapy	No	0.000	1(Ref.)			0.000	1(Ref.)		
	Yes		0.147	0.057	0.378		0.141	0.069	0.289
Radiotherapy	No	0.122	1(Ref.)			0.008	1(Ref.)		
	Yes		0.526	0.233	1.188		0.412	0.214	0.793
Endocrine therapy	No	0.001	1(Ref.)			0.000	1(Ref.)		
	Yes		0.193	0.072	0.517		0.264	0.148	0.468
Targeted therapy	No	0.286	1(Ref.)						
	Yes		0.559	0.192	1.626				

*Confounding factor. Abbreviation: OS: overall survival; GRIm score: Gustave Roussy Immune score; BMI: body mass index; ALT: alanine aminotransferase; AST: aspartate aminotransferase; LDH: lactate dehydrogenase; GGT: γ-glutamyl transpeptidase; ALB: albumin; TBIL: total bilirubin; DBIL: direct bilirubin; IBIL: indirect bilirubin; TP: total protein; G: globularproteins; PAB: prealbumin; CA153: cancer antigen 153; CEA: carcinoembryonic antigen; D-D: D-Dimer; FBG: fibrinogen; INR: international normalized ratio; NLR: neutrophil to lymphocyte ratio; ER: estrogen receptor; PR: progesterone receptor; HER2: human epidermal growth factor receptor 2; E-cad: E-cadherin.
