# Supplementary material for: The Gustave Roussy Immune score (GRIm score) as a novel prognostic score for early breast cancer patients: A real-world retrospective study
Source: Int J Med Sci. 2024 Oct 14;21(14):2640–54. doi: 10.7150/ijms.99724 (PMC11539385; doi:10.7150/ijms.99724)
Supplement: Supplementary file 1 — Supplementary figures and tables. [file ijmsv21p2640s1.pdf]

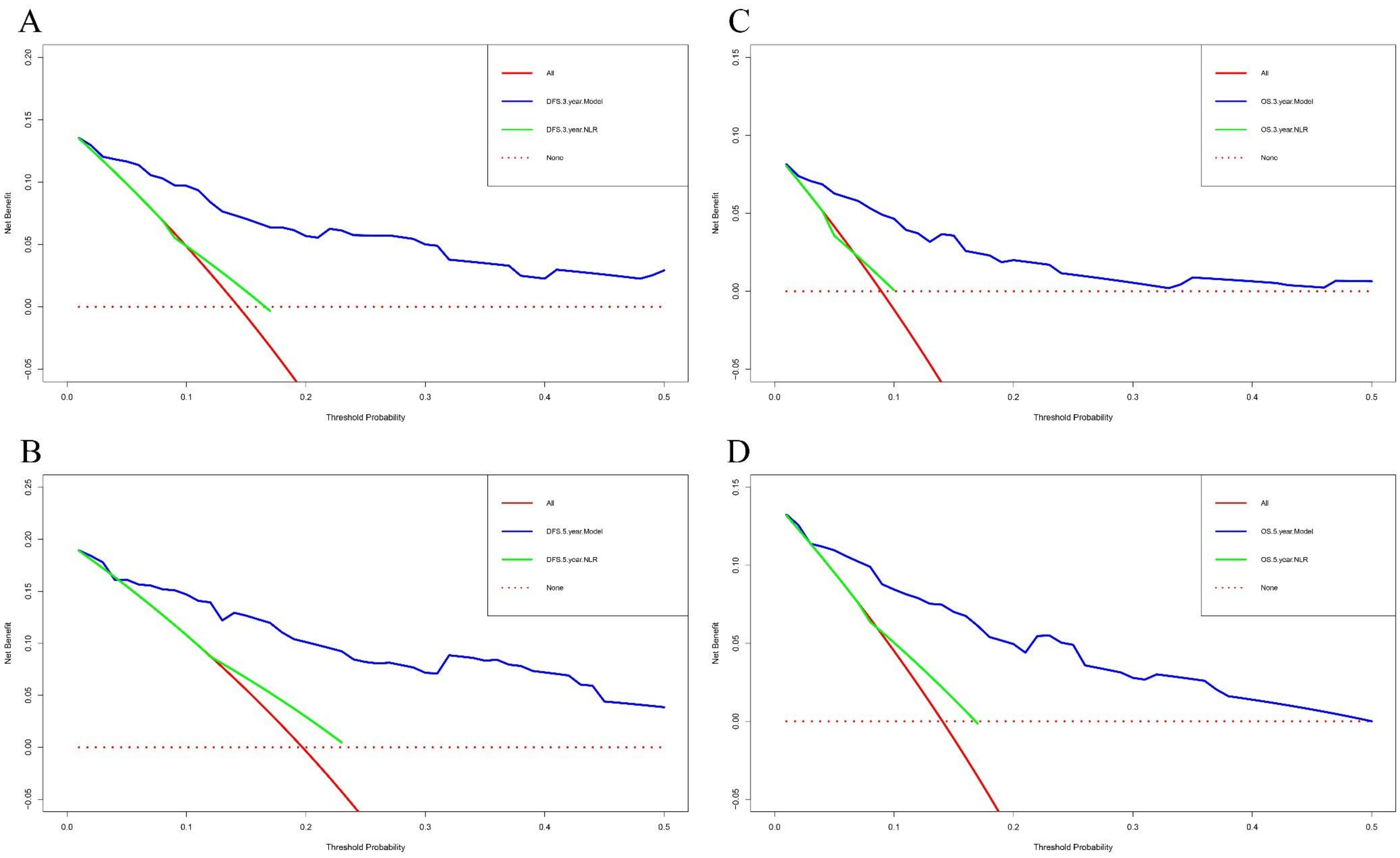
A
All
DFS.3.year.Model
DFS.3.year.NLR
None
Net Benefit
Threshold Probability
C
All
OS.3.year.Model
OS.3.year.NLR
None
Net Benefit
Threshold Probability
B
All
DFS.5.year.Model
DFS.5.year.NLR
None
Net Benefit
Threshold Probability
D
All
OS.5.year.Model
OS.5.year.NLR
None
Net Benefit
Threshold Probability

**Figure S1** Decision curve analyses (DCA) of nomogram and NLR prediction model for predicting the DFS and OS rates in 3- and 5-year. A) DCA of nomogram and GRIm score prediction model for predicting the DFS rates in 3-year; B) DCA of nomogram and GRIm score prediction model for predicting the DFS rates in 5-year; C) DCA of nomogram and GRIm score prediction model for predicting the OS rates in 3-year; D) DCA of nomogram and GRIm score prediction model for predicting the OS rates in 5-year.

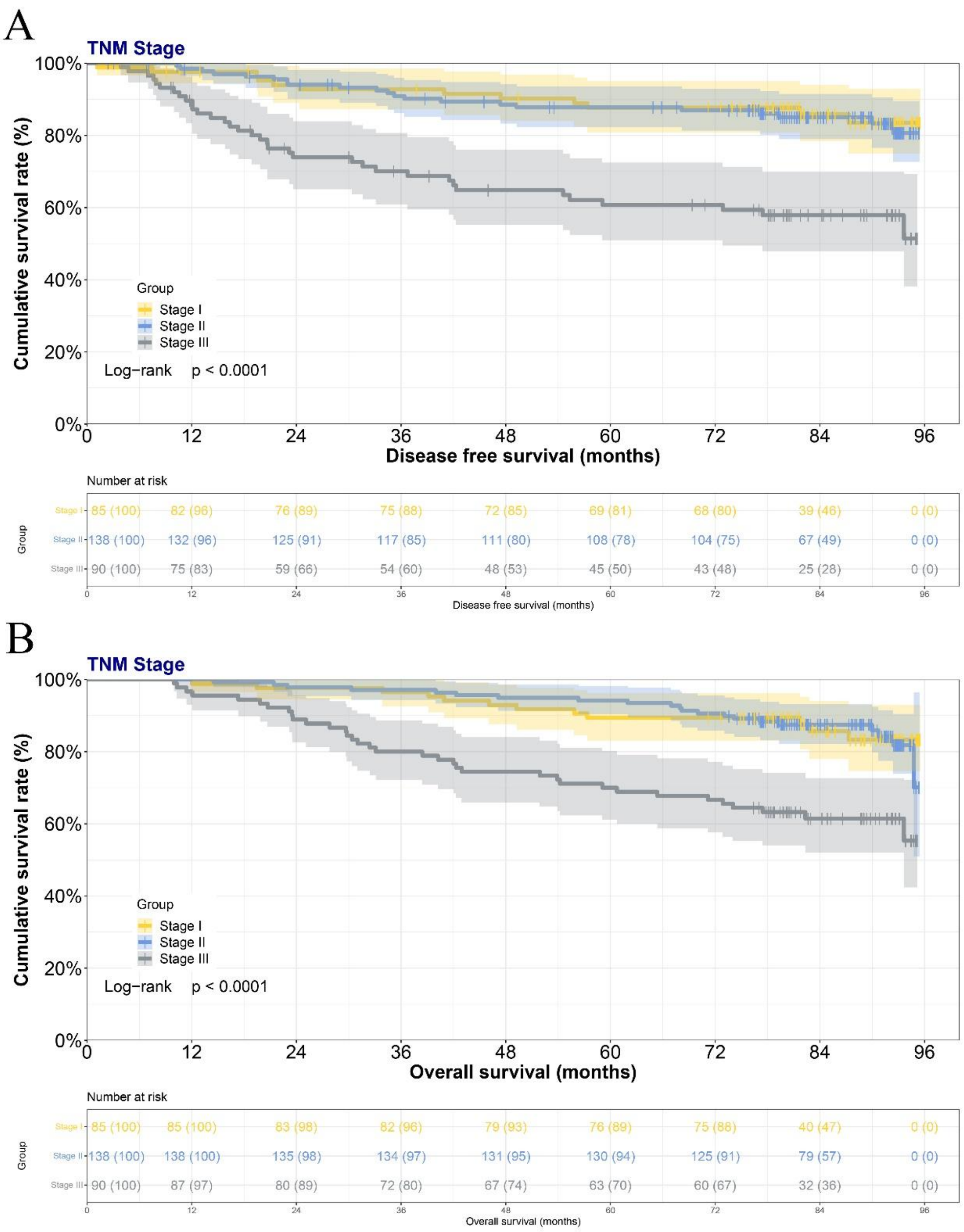


**Figure S2** The Kaplan-Meier survival curves illustrating the survival time among breast cancer patients, stratified based on the TNM stage. A) Kaplan-Meier survival curves illustrating the DFS among breast cancer patients, stratified based on the TNM stage; B) Kaplan-Meier survival curves illustrating the OS among breast cancer patients, stratified based on the TNM stage.

**Table S1** Correlation between GRIm score and organ metastasis

| Parameters | level | Overall | Low GRIm score | High GRIm score | p |
|---|---|---|---|---|---|
| n | | 313 | 81 | 232 | |
| Lung metastasis | No | 281 (89.8) | 74 (91.4) | 207 (89.2) | 0.739 |
| | Yes | 32 (10.2) | 7 (8.6) | 25 (10.8) | |
| Bone metastasis | No | 273 (87.2) | 72 (88.9) | 201 (86.6) | 0.742 |
| | Yes | 40 (12.8) | 9 (11.1) | 31 (13.4) | |
| Liver metastasis | No | 284 (90.7) | 74 (91.4) | 210 (90.5) | 0.998 |
| | Yes | 29 (9.3) | 7 (8.6) | 22 (9.5) | |
| Mediastinal metastasis | No | 299 (95.5) | 77 (95.1) | 222 (95.7) | 1.000 |
| | Yes | 14 (4.5) | 4 (4.9) | 10 (4.3) | |
| Brain metastasis | No | 297 (94.9) | 77 (95.1) | 220 (94.8) | 1.000 |
| | Yes | 16 (5.1) | 4 (4.9) | 12 (5.2) | |
| Pleural metastasis | No | 302 (96.5) | 78 (96.3) | 224 (96.6) | 1.000 |
| | Yes | 11 (3.5) | 3 (3.7) | 8 (3.4) | |
| Chest wall metastasis | No | 301 (96.2) | 75 (92.6) | 226 (97.4) | 0.108 |
| | Yes | 12 (3.8) | 6 (7.4) | 6 (2.6) | |
| Axillary metastasis | No | 154 (49.2) | 48 (59.3) | 106 (45.7) | 0.048 |
| | Yes | 159 (50.8) | 33 (40.7) | 126 (54.3) | |
| Clavicle metastasis | No | 264 (84.3) | 68 (84.0) | 196 (84.5) | 1.000 |
| | Yes | 49 (15.7) | 13 (16.0) | 36 (15.5) | |

Abbreviation: GRIm score: Gustave Roussy Immune score.

**Table S2** Comparison of performance of TNM stage with common hematological parameters

| Parameters | level | Overall | Stage I | Stage II | Stage III | p |
|---|---|---|---|---|---|---|
| n | | 313 | 85 | 138 | 90 | |
| GRIm score | Low | 81 (25.9) | 25 (29.4) | 38 (27.5) | 18 (20.0) | 0.305 |
| | High | 232 (74.1) | 60 (70.6) | 100 (72.5) | 72 (80.0) | |
| NLR | Low | 105 (33.5) | 29 (34.1) | 51 (37.0) | 25 (27.8) | 0.354 |
| | High | 208 (66.5) | 56 (65.9) | 87 (63.0) | 65 (72.2) | |
| ALT | <21 | 142 (45.4) | 44 (51.8) | 60 (43.5) | 38 (42.2) | 0.375 |
| | ≥21 | 171 (54.6) | 41 (48.2) | 78 (56.5) | 52 (57.8) | |
| AST | <23 | 147 (47.0) | 41 (48.2) | 67 (48.6) | 39 (43.3) | 0.715 |
| | ≥23 | 166 (53.0) | 44 (51.8) | 71 (51.4) | 51 (56.7) | |
| AST/ALT | <1.44 | 155 (49.5) | 73 (85.9) | 117 (84.8) | 77 (85.6) | 0.972 |
| | ≥1.44 | 158 (50.5) | 12 (14.1) | 21 (15.2) | 13 (14.4) | |
| LDH | <170 | 156 (49.8) | 52 (61.2) | 63 (45.7) | 41 (45.6) | 0.050 |
| | ≥170 | 157 (50.2) | 33 (38.8) | 75 (54.3) | 49 (54.4) | |
| ALB | <45 | 145 (46.3) | 41 (48.2) | 63 (45.7) | 41 (45.6) | 0.918 |
| | ≥45 | 168 (53.7) | 44 (51.8) | 75 (54.3) | 49 (54.4) | |
| TBIL | <12.45 | 156 (49.8) | 55 (64.7) | 66 (47.8) | 35 (38.9) | 0.002 |
| | ≥12.45 | 157 (50.2) | 30 (35.3) | 72 (52.2) | 55 (61.1) | |
| CA153 | <9.82 | 156 (49.8) | 52 (61.2) | 74 (53.6) | 30 (33.3) | 0.001 |
| | ≥9.82 | 157 (50.2) | 33 (38.8) | 64 (46.4) | 60 (66.7) | |
| CEA | <1.49 | 156 (49.8) | 50 (58.8) | 74 (53.6) | 32 (35.6) | 0.004 |
| | ≥1.49 | 157 (50.2) | 35 (41.2) | 64 (46.4) | 58 (64.4) | |
| D-D | <0.25 | 151 (48.2) | 42 (49.4) | 69 (50.0) | 40 (44.4) | 0.692 |
| | ≥0.25 | 162 (51.8) | 43 (50.6) | 69 (50.0) | 50 (55.6) | |
| FBG | <2.6 | 153 (48.9) | 39 (45.9) | 69 (50.0) | 45 (50.0) | 0.811 |
| | ≥2.6 | 160 (51.1) | 46 (54.1) | 69 (50.0) | 45 (50.0) | |
| White blood cell | <5.45 | 156 (49.8) | 50 (58.8) | 63 (45.7) | 43 (47.8) | 0.145 |

| | | | | | | |
|---|---|---|---|---|---|---|
| | ≥5.45 | 157 (50.2) | 35 (41.2) | 75 (54.3) | 47 (52.2) | |
| Neutrophil | <3.23 | 155 (49.5) | 47 (55.3) | 67 (48.6) | 41 (45.6) | 0.417 |
| | ≥3.23 | 158 (50.5) | 38 (44.7) | 71 (51.4) | 49 (54.4) | |
| Lymphocyte | <1.70 | 154 (49.2) | 42 (49.4) | 62 (44.9) | 50 (55.6) | 0.292 |
| | ≥1.70 | 159 (50.8) | 43 (50.6) | 76 (55.1) | 40 (44.4) | |
| Monocyte | <0.35 | 151 (48.2) | 47 (55.3) | 55 (39.9) | 49 (54.4) | 0.031 |
| | ≥0.35 | 162 (51.8) | 38 (44.7) | 83 (60.1) | 41 (45.6) | |
| Platelet | <233 | 153 (48.9) | 40 (47.1) | 60 (43.5) | 53 (58.9) | 0.070 |
| | ≥233 | 160 (51.1) | 45 (52.9) | 78 (56.5) | 37 (41.1) | |

Abbreviation: GRIm score: Gustave Roussy Immune score; NLR: neutrophil to lymphocyte ratio; ALT: alanine aminotransferase; AST: aspartate aminotransferase; LDH: lactate dehydrogenase; ALB: albumin; TBIL: total bilirubin; CA153: cancer antigen 153; CEA: carcinoembryonic antigen; D-D: D-Dimer; FBG: fibrinogen.

**Table S3** Comparison of prognostic effect of the GRIm score and its components by different TNM stage

| Parameters | | Stage I | | | | Stage II | | | | Stage III | | | |
|---|---|---|---|---|---|---|---|---|---|---|---|---|---|
| | level | Overall | Low GRIm score | High GRIm score | p | Overall | Low GRIm score | High GRIm score | p | Overall | Low GRIm score | High GRIm score | p |
| n | | 85 | 25 | 60 | | 138 | 38 | 100 | | 90 | 18 | 72 | |
| NLR | Low | 29 (34.1) | 25 (100.0) | 4 (6.7) | <0.001 | 51 (37.0) | 38 (100.0) | 13 (13.0) | <0.001 | 25 (27.8) | 17 (94.4) | 8 (11.1) | <0.001 |
| | High | 56 (65.9) | 0 (0.0) | 56 (93.3) | | 87 (63.0) | 0 (0.0) | 87 (87.0) | | 65 (72.2) | 1 (5.6) | 64 (88.9) | |
| ALT | <21 | 44 (51.8) | 13 (52.0) | 31 (51.7) | 1.000 | 60 (43.5) | 16 (42.1) | 44 (44.0) | 0.993 | 38 (42.2) | 4 (22.2) | 34 (47.2) | 0.098 |
| | ≥21 | 41 (48.2) | 12 (48.0) | 29 (48.3) | | 78 (56.5) | 22 (57.9) | 56 (56.0) | | 52 (57.8) | 14 (77.8) | 38 (52.8) | |
| AST | <23 | 41 (48.2) | 10 (40.0) | 31 (51.7) | 0.458 | 67 (48.6) | 19 (50.0) | 48 (48.0) | 0.985 | 39 (43.3) | 7 (38.9) | 32 (44.4) | 0.873 |
| | ≥23 | 44 (51.8) | 15 (60.0) | 29 (48.3) | | 71 (51.4) | 19 (50.0) | 52 (52.0) | | 51 (56.7) | 11 (61.1) | 40 (55.6) | |
| AST/ALT | <1.1 | 38 (44.7) | 13 (52.0) | 25 (41.7) | 0.526 | 70 (50.7) | 20 (52.6) | 50 (50.0) | 0.932 | 47 (52.2) | 13 (72.2) | 34 (47.2) | 0.102 |
| | ≥1.1 | 47 (55.3) | 12 (48.0) | 35 (58.3) | | 68 (49.3) | 18 (47.4) | 50 (50.0) | | 43 (47.8) | 5 (27.8) | 38 (52.8) | |
| LDH | <170 | 52 (61.2) | 13 (52.0) | 39 (65.0) | 0.381 | 63 (45.7) | 16 (42.1) | 47 (47.0) | 0.746 | 41 (45.6) | 9 (50.0) | 32 (44.4) | 0.874 |
| | ≥170 | 33 (38.8) | 12 (48.0) | 21 (35.0) | | 75 (54.3) | 22 (57.9) | 53 (53.0) | | 49 (54.4) | 9 (50.0) | 40 (55.6) | |
| ALB | <45 | 41 (48.2) | 12 (48.0) | 29 (48.3) | 1.000 | 63 (45.7) | 20 (52.6) | 43 (43.0) | 0.410 | 41 (45.6) | 11 (61.1) | 30 (41.7) | 0.224 |
| | ≥45 | 44 (51.8) | 13 (52.0) | 31 (51.7) | | 75 (54.3) | 18 (47.4) | 57 (57.0) | | 49 (54.4) | 7 (38.9) | 42 (58.3) | |
| TBIL | <12.45 | 55 (64.7) | 16 (64.0) | 39 (65.0) | 1.000 | 66 (47.8) | 21 (55.3) | 45 (45.0) | 0.375 | 35 (38.9) | 7 (38.9) | 28 (38.9) | 1.000 |
| | ≥12.45 | 30 (35.3) | 9 (36.0) | 21 (35.0) | | 72 (52.2) | 17 (44.7) | 55 (55.0) | | 55 (61.1) | 11 (61.1) | 44 (61.1) | |
| CA153 | <9.82 | 52 (61.2) | 16 (64.0) | 36 (60.0) | 0.920 | 74 (53.6) | 22 (57.9) | 52 (52.0) | 0.668 | 30 (33.3) | 6 (33.3) | 24 (33.3) | 1.000 |
| | ≥9.82 | 33 (38.8) | 9 (36.0) | 24 (40.0) | | 64 (46.4) | 16 (42.1) | 48 (48.0) | | 60 (66.7) | 12 (66.7) | 48 (66.7) | |
| CEA | <1.49 | 50 (58.8) | 14 (56.0) | 36 (60.0) | 0.921 | 74 (53.6) | 17 (44.7) | 57 (57.0) | 0.272 | 32 (35.6) | 4 (22.2) | 28 (38.9) | 0.296 |
| | ≥1.49 | 35 (41.2) | 11 (44.0) | 24 (40.0) | | 64 (46.4) | 21 (55.3) | 43 (43.0) | | 58 (64.4) | 14 (77.8) | 44 (61.1) | |
| D-D | <0.25 | 42 (49.4) | 15 (60.0) | 27 (45.0) | 0.307 | 69 (50.0) | 17 (44.7) | 52 (52.0) | 0.568 | 40 (44.4) | 9 (50.0) | 31 (43.1) | 0.791 |
| | ≥0.25 | 43 (50.6) | 10 (40.0) | 33 (55.0) | | 69 (50.0) | 21 (55.3) | 48 (48.0) | | 50 (55.6) | 9 (50.0) | 41 (56.9) | |
| FBG | <2.6 | 39 (45.9) | 15 (60.0) | 24 (40.0) | 0.148 | 69 (50.0) | 22 (57.9) | 47 (47.0) | 0.341 | 45 (50.0) | 11 (61.1) | 34 (47.2) | 0.429 |

| | | | | | | | | | | | | | |
|---|---|---|---|---|---|---|---|---|---|---|---|---|---|
| | ≥2.6 | 46 (54.1) | 10 (40.0) | 36 (60.0) | | 69 (50.0) | 16 (42.1) | 53 (53.0) | | 45 (50.0) | 7 (38.9) | 38 (52.8) | |
| White blood cell | <5.45 | 50 (58.8) | 17 (68.0) | 33 (55.0) | 0.386 | 63 (45.7) | 28 (73.7) | 35 (35.0) | <0.001 | 43 (47.8) | 8 (44.4) | 35 (48.6) | 0.958 |
| | ≥5.45 | 35 (41.2) | 8 (32.0) | 27 (45.0) | | 75 (54.3) | 10 (26.3) | 65 (65.0) | | 47 (52.2) | 10 (55.6) | 37 (51.4) | |
| Neutrophil | <3.23 | 47 (55.3) | 21 (84.0) | 26 (43.3) | 0.001 | 67 (48.6) | 32 (84.2) | 35 (35.0) | <0.001 | 41 (45.6) | 15 (83.3) | 26 (36.1) | 0.001 |
| | ≥3.23 | 38 (44.7) | 4 (16.0) | 34 (56.7) | | 71 (51.4) | 6 (15.8) | 65 (65.0) | | 49 (54.4) | 3 (16.7) | 46 (63.9) | |
| Lymphocyte | <1.70 | 42 (49.4) | 4 (16.0) | 38 (63.3) | <0.001 | 62 (44.9) | 10 (26.3) | 52 (52.0) | 0.012 | 50 (55.6) | 5 (27.8) | 45 (62.5) | 0.017 |
| | ≥1.70 | 43 (50.6) | 21 (84.0) | 22 (36.7) | | 76 (55.1) | 28 (73.7) | 48 (48.0) | | 40 (44.4) | 13 (72.2) | 27 (37.5) | |
| Monocyte | <0.35 | 47 (55.3) | 13 (52.0) | 34 (56.7) | 0.877 | 55 (39.9) | 21 (55.3) | 34 (34.0) | 0.037 | 49 (54.4) | 14 (77.8) | 35 (48.6) | 0.050 |
| | ≥0.35 | 38 (44.7) | 12 (48.0) | 26 (43.3) | | 83 (60.1) | 17 (44.7) | 66 (66.0) | | 41 (45.6) | 4 (22.2) | 37 (51.4) | |
| Platelet | <233 | 40 (47.1) | 13 (52.0) | 27 (45.0) | 0.726 | 60 (43.5) | 15 (39.5) | 45 (45.0) | 0.694 | 53 (58.9) | 9 (50.0) | 44 (61.1) | 0.556 |
| | ≥233 | 45 (52.9) | 12 (48.0) | 33 (55.0) | | 78 (56.5) | 23 (60.5) | 55 (55.0) | | 37 (41.1) | 9 (50.0) | 28 (38.9) | |

Abbreviation: GRIm score: Gustave Roussy Immune score; NLR: neutrophil to lymphocyte ratio; ALT: alanine aminotransferase; AST: aspartate aminotransferase; LDH: lactate dehydrogenase; ALB: albumin; TBIL: total bilirubin; CA153: cancer antigen 153; CEA: carcinoembryonic antigen; D-D: D-Dimer; FBG: fibrinogen.
